# Sexually Dimorphic and Sex-Independent Left-Right Asymmetries in Chicken Embryonic Gonads

**DOI:** 10.1371/journal.pone.0069893

**Published:** 2013-07-19

**Authors:** Sittipon Intarapat, Claudio D. Stern

**Affiliations:** 1 Department of Anatomy, Faculty of Science, Mahidol University, Bangkok, Thailand; 2 Department of Cell and Developmental Biology and UCL Centre for Stem Cells and Regenerative Medicine, University College London, London, United Kingdom; National University of Singapore, Singapore

## Abstract

Female birds develop asymmetric gonads: a functional ovary develops on the left, whereas the right gonad regresses. In males, however, testes develop on both sides. We examined the distribution of germ cells using *Vasa/Cvh* as a marker. Expression is asymmetric in both sexes: at stage 35 the left gonad contains significantly more germ cells than the right. A similar expression pattern is seen for expression of *ERNI* (*Ens1*), a gene expressed in chick embryonic stem cells while they self-renew, but downregulated upon differentiation. Other pluripotency-associated markers (*PouV/Oct3/4*, *Nanog* and *Sox2*) also show asymmetric expression (more expressing cells on the left) in both sexes, but this asymmetry is at least partly due to expression in stromal cells of the developing gonad, and the pattern is different for all the genes. Therefore germ cell and pluripotency-associated genes show both sex-dependent and independent left-right asymmetry and a complex pattern of expression.

## Introduction

Unlike mammals, which have apparently symmetric gonads, most female bird species develop asymmetrically, generating a functional ovary only on the left side, whereas males develop bilateral testes [1]. Before sexual differentiation (the “indifferent stage”), there is no detectable morphological asymmetry between left and right embryonic gonads in either sex. Morphological differences in embryonic gonads appear after sexual differentiation; male embryos (which are the homogametic sex, ZZ) develop bilateral testes, while female embryos (heterogametic, ZW) develop a functional left ovary and the right ovary regresses [2].

The gonads of both sexes contain two layers, cortex and medulla [2], [3]. These change and become sexually dimorphic during gonadal differentiation. Embryonic testes exhibit greater medullary development by the appearance of testicular cords containing the male germ cells, supporting Sertoli cells inside and hormone-producing Leydig cells outside the cords. On the other hand, the ovary exhibits greater cortical development, and female germ cells locate in this layer [4]. Early differences between male and female embryos are thought to include a greater number and size of female germ cells at an earlier stage than in males [5], based on the localization of glycogen granules in germ cells by PAS staining [6], [7].

Several genes underlie sexual differentiation and lie near the top of a genetic hierarchy governing sex specific differences. Among the genes that differ between male and female embryos at an early stage, *DMRT1* [8], [9] and *Sox9* [10], [11] are expressed in male (ZZ) embryos, whereas *HINTW* [12], [13], *FET1* [14] and *FOXL2* [15] are expressed in female (ZW) embryos. Aromatase, a key enzyme for converting testosterone into oestrogen, is expressed only in female gonads [16], [17].

In addition to differential expression of molecular components related to sexual differentiation and function, a few genes have been described to display left-right differences in expression. Not surprisingly, given its role as a highly conserved determinant of left-sidedness in many organ systems [18], [19], [20], [21], [22], [23], [24], [25], [26], *PITX2* is expressed in the left female gonad, where its functions include stimulation of gonadal cell proliferation and morphogenesis [27], [28], [29]. *Bmp7* is also expressed asymmetrically, showing different patterns on the left and right gonadal primordia at the beginning of genital ridge formation (sex indifferent stage), but in a sex-specific way after sexual differentiation, in ovarian mesenchyme [30]. BMPs have been shown to play a role in left-right asymmetry in earlier development, and it is conceivable that this asymmetric expression relates to a similar function in gonadal development. Finally, estrogen receptor alpha (ERα) is expressed in the left but not the right cortex of both sexes [17], [31]; the significance of this asymmetry (especially in the male) is unknown.

Cell lines derived from pre-primitive streak stage embryos (“chick ES cells”) can contribute to all somatic lineages but not to the germ line [32], [33], [34] whereas PGCs obtained either from the circulation or from the gonads are truly pluripotent [35], [36]. The present study arose from an attempt to identify the latter cells in the gonad, in vivo, to aid the development of more efficient methods for their isolation and to begin to characterise them molecularly. We used the expression of the chick homologue of the germ cell marker *Vasa* (*Cvh*) to identify primordial germ cells and gonocytes, since this gene is expressed only in germ cells at all stages of development and also appears to be both necessary and sufficient to confer germ line competence to cells, including chick ES cells [37]. We also examined the expression of the pluripotency-associated genes *Nanog*, *PouV* (*Oct3/4*) and *Sox2* [38], [39], [40] and of *ERNI*, a gene originally identified as an early response to neural induction [41] and also found to be expressed in chick ES cells while in the self-renewing, undifferentiated state but downregulated upon differentiation [42], [43]. Consistent with a previous report [29], *Cvh*-positive PGCs are located preferentially in the left gonad of both sexes. More surprisingly, however, asymmetry of expression is seen for all genes, but the patterns are not identical. Some of the asymmetries can be related to the presence of germ cell precursors but some are clearly independent, revealing a greater degree of complexity of left-right asymmetric molecular components in the gonads of both sexes than hitherto suspected.

## Results

### Asymmetric Distribution of Germ Cells in the Gonads of both Sexes

**Figure 1:**
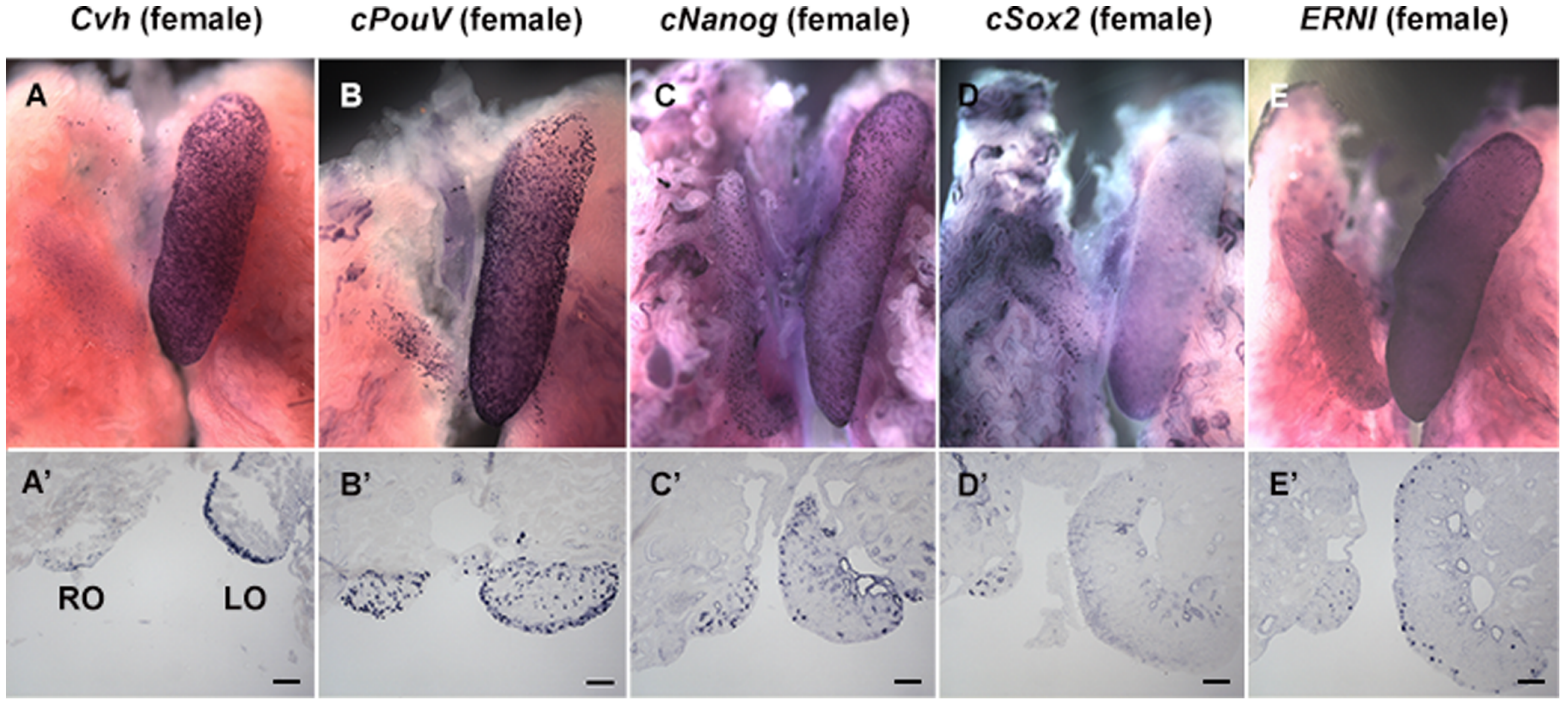
Left-right asymmetric gene expression in female embryonic gonads: *Cvh* (A), *cPouV* (B), *cNanog* (C), *cSox2* (D) and *ERNI* (E) positive cells are expressed in both left and right testes. Testicular sections exhibit germ cells, *Cvh* (A’) and *cPouV* (B’), *cNanog* (C’), *cSox2* (D’) and *ERNI* (E’) positive cells. Abbreviations: RT = Right testes, LT = Left testes. (Scale bar = 50 µm).

As early as 1935, Witschi suggested that the left embryonic ovary contains more germ cells than the right, consistent with the obvious difference in development of these female organs (the right ovary does not develop to adulthood) [44]. To visualise germ cells in the gonads we performed in situ hybridisation in whole mounts and sections of gonads of female and male embryos at stage HH 35 (9 days’ incubation; [45]) for the germ cell marker, *Cvh* (Fig. 1A, 2A). As expected, sections through left and right ovaries revealed significant differences in *Cvh*-positive cell numbers: 61±30 per transverse section on the left and 2±4 on the right; *p*<0.001, n = 64 sections through 3 embryos; Fig. 3; see also Table 1). However, left-right differences were also found in the male: the average number of germ cells expressing *Cvh* in left and right male gonads was 21±16 and 11±11 respectively (*p*<0.001, n = 78 sections in 3 embryos, Fig. 3).

**Figure 2:**
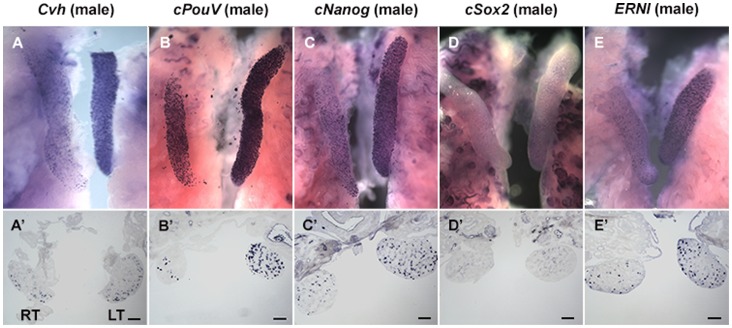
Left-right asymmetric gene expression in male embryonic gonads: *Cvh* (A), *cPouV* (B), *cNanog* (C), *cSox2* (D) and *ERNI* (E) positive cells are expressed in both left and right ovaries. Ovarian sections exhibit germ cells, *Cvh* (A’) and *cPouV* (B’), *cNanog* (C’), *cSox2* (D’) and *ERNI* (E’) positive cells. Abbreviations: RO = Right ovary, LO = Left ovary. (Scale bar = 50 µm).

**Figure 3:**
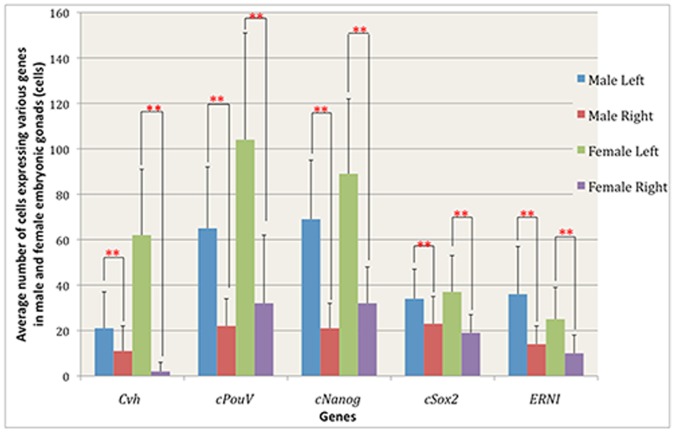
Quantification of cells expressing various genes in male and female embryonic gonads.

**Table 1:** Summary of samples used in this study and cell expressing various genes in female-male and left-right embryonic gonads.

Gene	Number ofembryos (*n*)	Number ofsections (total)	Expressing cells inleft gonad sections	Expressing cells inright gonad sections	Expressing cells in left cortex	Expressing cells in right cortex	Expressing cells in left medulla	Expressing cells in right medulla
*Cvh*	3F/3M	64F/78M	F(61±30)**a_1_, M(21±16)**a_2_	F(2±4)**a_1,_ M(11±11)**a_2_	F(54±34)**b_1_, M(5±2)	F(3±3)**b_1,_ M(5±4)	F(9±14), M(16±9)	F(12±8), M(16±7)
*cPouV*	3F/3M	34F/66M	F (104±47)**a_1,_ M(65±27)**a_2_	F(32±30)**a_1,_ M(22±12)**a_2_	F(70±33)**b_1,_ M(8±5)*b_2_	F(32±29)**b_1,_ M(4±3)*b_2_	F(38±29)**c_1,_ M(49±24)**c_2_	F(8±7)**c_1,_ M(12±12)**c_2_
*cNanog*	3F/3M	62F/73M	F(89±33)**a_1,_ M(69±26)**a_2_	F(32±16)**a_1,_ M(21±11)**a_2_	F(45±21)**b_1,_ M(16±8)**b_2_	F(13±9)**b_1,_ M(5±2)**b_2_	F(43±15)**c_1_, M(50±23)**c_2_	F(21±13)**c_1_, M(17±9)**c_2_
*cSox2*	3F/4M	53F/101M	F(37±16)**a_1_, M(34±13)**a_2_	F(19±8)**a_1_, M(23±12)**a_2_	F(11±22), M(7±6)	F(1±2), M(6±4)	F(22±6)**c_1_, M(35±13)	F(13±4)**c_1_, M(28±12)
*ERNI*	3F/3M	63F/50M	F(25±14)**a_1_, M(36±21)**a_2_	F(10±8)**a_1_, M(14±8)**a_2_	F(28±9)**b_1_, M(8±4)**b_2_	F(9±5)**b_1_, M(3±3)**b_2_	F(9±4), M(34±18)**c_2_	F(8±6), M(15±9)**c_2_

F: Female; M: Male (** *p*-value <0.01, * *p*-value <0.05, Student’s *t*-test).

a_1_: a comparison between female left and right gonad sections; b_1_: a comparison between female left and right cortices; c_1_: a comparison between female left and right medullae; a_2_: a comparison between male left and right gonad sections; b_2_: a comparison between male left and right cortices; c_2_: a comparison between male left and right medullae.

**Figure 4:**
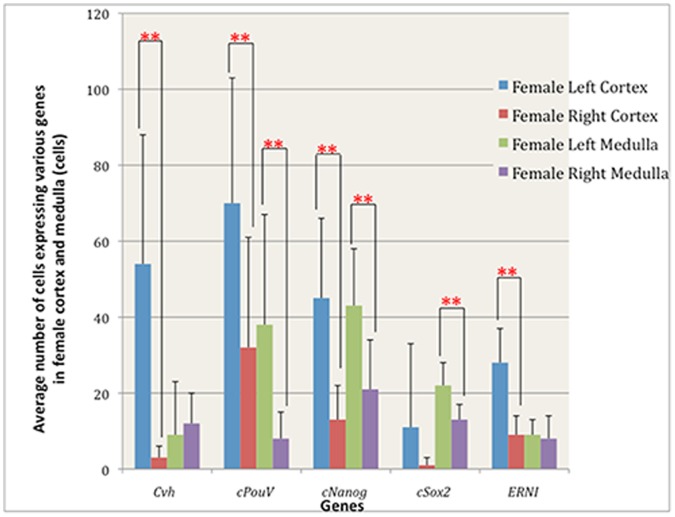
Quantification of cells expressing various genes in female cortex and medulla.

**Figure 5:**
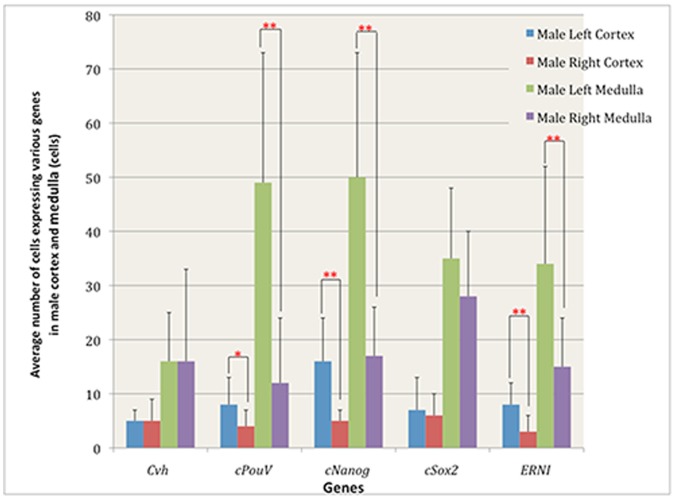
Quantification of cells expressing various genes in male cortex and medulla.

Almost all the female germ cells are located in the ovarian cortex (Fig. 1A’): 54±34 on the left and 3±3 on the right cortex; *p = *0.002, n = 9 sections in 3 embryos; Fig. 4), and 9±14 on the left and 12±8 on the right medulla (no significant left/right difference; p = 0.64; Fig. 4, Table 1). In male, cells expressing *Cvh* are located in both the cortex and the medulla (Fig. 2A’): an average of 5±2 and 5±4 cells were found in the cortices of left and right gonadal sections (Fig. 5, Table 1), while 16±9 and 16±17 were found in the left and right medulla respectively (n = 9 sections, 3 embryos; no significant left/right difference in either cortex or medulla; p>0.9 Fig. 5, Table 1).

These results reveal left-right differences in germ cell distribution in the gonads of embryos of both sexes: the left gonad contains a greater number of *Cvh*-positive cells than the right, confirming a previous report [29]. However the asymmetry is more pronounced in females than in males.

### Asymmetric Expression of Pluripotency-related Genes *PouV, Nanog* and *Sox2* in the Gonads of Both Sexes

Next, we examined embryonic ovaries and testes for expression of three pluripotency-associated genes: *PouV* (* = Oct3/4*; Fig. 1B, 2B), *Nanog* (Fig. 1C, 2C) and *Sox2* (Fig. 1D, 2D).

The average number of cells expressing *PouV* in the left female gonad was significantly higher than in the right (respectively 104±47 and 32±30; *p*<0.001, n = 34 sections through 3 embryos; Fig. 3). In the ovarian cortex there were 70±33 and 32±29 for left and right respectively (*p* = 0.017, n = 9 sections, 3 embryos; Fig. 4, Table 1) whereas in the medulla 38±29 and 8±7, *p* = 0.015 were counted on the left and right respectively; Fig. 4, Table 1). Given that females have very few germ cells in the medulla at this stage (see above), the majority of these *PouV* expressing ovarian medullary cells (Fig. 1B’) are likely to be stromal cells.

The average number of cells expressing *PouV* in left and right male gonads was 65±27 and 22±12 per section, respectively (*p*<0.001, n = 66 sections, 3 embryos); Fig. 3, Table 1). Very few *PouV* expressing cells were found in the testicular cortex, slightly more on the left than the right: 8±5 and 4±3 per section respectively, (*p* = 0.023; n = 9 sections, 3 embryos; Fig. 5, Table 1). The testicular medulla showed more marked left-right differences: 49±24 for the left and 12±12, for the right, *p* = 0.0014; Fig. 5, Table 1).

*Nanog* (Fig. 1C’) has a pattern of localization similar to that of *PouV*. Expressing cells were detected in both cortex and medulla in the left and the right female gonads (Fig. 1B’) with significant left-right differences: 89±33 per section on the left and 32±16 per section on the right (*p<*0.001, n = 62 sections, 3 embryos; Fig. 3, Table 1). The average number of cells expressing *Nanog* in left and right male gonads was 69±26 and 21±11 per section (*p*<0.001, n = 73 sections, 3 embryos; Fig. 3, Table 1).

In ovarian cortex, there were 45±21 *cNanog*-expressing cells on the left and 13±9 on the right (*p* = 0.0017, n = 9 sections, 3 embryos; Fig. 4, Table 1). In the medulla, 43±15 were counted on the left and 21±13 on the right (*p = *0.004, (n = 3); Fig. 4, Table 1). In testicular cortex 16±8 were observed on the left and 5±2 on the right (*p* = 0.0027, n = 9 sections, 3 embryos, Fig. 5, Table 1), whereas the medulla contained 50±23 on the left and 17±9 on the right (*p* = 0.002, Fig. 5, Table 1).

Therefore again the majority of the medullary ovarian cells expressing *Nanog* is unlikely to correspond to germ cells. Moreover, there appear to be more *Nanog* expressing cells in both cortex and medulla of both male and female gonads than *Cvh*-expressing cells, suggesting that the left-right differences in expression of these genes at least includes a contribution from stromal cells.

In female, the average number of cells expressing *Sox2* in the left and the right gonads was 37±16 and 19±8 respectively (*p*<0.001, n = 53 sections, 3 embryos; Fig. 3, Table 1). The ovarian cortex contained 11±22 on the left and 1±2 on the right per gonadal section (n = 9 sections, 3 embryos; Fig. 4, Table 1) whereas the left and right ovarian medulla contained 22±6 and 13±4 respectively (*p* = 0.002, n = 9 sections, 3 embryos; Fig. 4, Table 1). In the male, the average number of cells expressing *Sox2* in the left gonad was significantly higher than that on the right (34±13 and 23±12 respectively; *p*<0.001, n = 101 sections, 4 embryos; Fig. 3, Table 1). The testicular cortex contained 7±6 and 6±4 per gonadal sections on the left and right respectively (n = 12 sections, 4 embryos; Fig. 5, Table 1), whereas the medulla contained 35±13 on the left and 28±12 on the right (Fig. 5, Table 1). Thus, very few *Sox2* expressing cells were observed in the cortex in testicular sections (Fig. 2D’) while *Sox2* expression was detected in both cortex and medulla in ovarian sections (Fig. 1D’).

These results reveal that there is little or no correlation between *Sox2* expression and the distribution of *Cvh*-positive germ cells. Moreover the morphology of *Sox2* expressing cells is more akin to tubular cells than germ cells especially in the ovarian medulla (eg. see Fig. 1D’). However, significant left-right differences are observed for *Sox2* expression in both sexes, although this is less marked than for the other genes studied here (eg. See Fig. 3, Table 1).

### *ERNI* is Asymmetrically Expressed in the Gonads of Both Sexes

Embryonic ovaries and testes also express *ERNI* [41], a gene whose expression relates to the self-renewing, proliferating state in chick ES cells ( = *Ens1*; [42], [43]); see Fig. 1E, 2E). In female, the average number of cells expressing *ERNI* in the left and right gonads was 25±14 and 10±8 respectively (*p*<0.001, n = 63 sections, 3 embryos; Fig. 3, Table 1). In male, the average number of cells expressing *ERNI* in the left was also significantly higher than that in the right gonads (36±21 and 14±8; *p*<0.001, n = 50 sections, 3 embryos; Fig. 3, Table 1).

*ERNI* was detected in cortex and a few cells in the medulla in ovarian sections (Fig. 1E’), while in testicular sections, *ERNI* expressing cells are found in both cortex and medulla (Fig. 2E’). In ovarian cortex, 28±9 were counted on the left and 9±5 on the right (*p*<0.001, n = 9 sections, 3 embryos; Fig. 4, Table 1) whereas the medulla contained 9±4 on the left and 8±6 on the right (Fig. 4, Table 1). In the left and right testicular cortex 8±4 and 3±3 cells were counted respectively (Fig. 5, Table 1) whereas the testicular medulla contained 34±18 on the left and 15±9 on the right (*p* = 0.0147, n = 9 sections, 3 embryos; Fig. 5, Table 1). Thus, although *ERNI* also seems to be expressed in stromal cells in addition to germ cells, its expression reflects the distribution of *Cvh*-expressing cells in embryos of both sexes more closely than do the other pluripotency-associated genes.

## Discussion

A distinctive feature of gonadal development in the chick is that female embryos develop gonads asymmetrically: only the left side forms a functional ovary while the right side regresses [2]. The molecular mechanisms underlying asymmetric development of female embryonic chick gonads are still unclear, apart from the involvement of the transcription factor Pitx2 [27], [28], [29], whose asymmetric expression seems to underlie the asymmetric development of many, if not all organ systems [18], [19], [26].

Left-right patterning plays important roles for internal organ formation, positioning and embryonic turning [18], [19], [20], [21], [22], [23], [24], [26], [46]. The process is regulated by genes encoding transcription factors and secreted growth factors, but there are important differences among different vertebrates in terms of which specific genes are involved [19], [21]. To date only two main players have been found to be conserved in all vertebrates: Pitx2 and Nodal [18], [19], [20], [26]. The latter is a secreted protein of the TGFβ superfamily. PITX2 is a homeobox-containing transcription factor with a bicoid-type homeodomain. Both are involved in establishing L-R asymmetry through their expression in the left lateral plate mesoderm and later in a number of organs such as the heart and head [18], [47], [48]. *Pitx2*-knockout mice have abnormalities of internal organ asymmetry [49], [50], showing that this gene plays an essential role in controlling laterality in mice. Consistent with this, *PITX2* was reported to play a role in ovarian asymmetric development in female embryos; it is preferentially expressed in the left gonad, where it may regulate gonadal cell proliferation and morphogenesis [27], [28], [29].

Other previous studies also reported asymmetric gonad development in chick embryos, suggesting that 70% of PGCs are found on the left side of female gonads [44], [51], [52], [53]. It has been proposed that the left presumptive gonad secretes chemotactic factors at a higher level than the right, which was proposed to be involved in attracting migrating PGCs as well as regulating their mitotic activity [54]. Although one study has reported a similar asymmetry for male PGCs [29], our study reveals more complex asymmetry of gene expression in both sexes, not only for PGC markers but also for pluripotency markers in both PGCs and stromal cells.

That *Cvh* expression is higher in the left gonad of both sexes indicates that asymmetric germ cell distribution is not entirely related to the later asymmetry of gonadal differentiation, which is so marked in the female. It would be interesting to investigate this in mature adults to determine whether the left-right differences in germ cell numbers persist and eventually translate into asymmetry in the rate of sperm production in roosters.

The present study also provides novel information about the expression of genes associated with pluripotency in embryonic gonads of both sexes and between left and right gonads. For all four genes studied (*cPouV*, *cNanog*, *cSox2* and *ERNI*), the number of cells expressing are significantly higher on the left than the right gonads in embryos of both sexes. However the numbers of cells expressing the markers are not identical to each other – there are more cells expressing the first two than there are *Cvh*-expressing cells and cells expressing the latter two factors. The distribution of the cells expressing the various genes is also different from the distribution of PGCs. *ERNI* correlates best with *Cvh* expression, whereas the remaining factors are also expressed in cells that are unlikely to be PGCs yet show left-right asymmetry of expression. This suggests that stromal cells express these genes asymmetrically. The functional significance of this complex expression pattern is unclear.

At stage HH 35, embryonic gonads are well into their differentiation into testes and ovaries. Studies in humans have reported that both fetal testicular and ovarian germ cells express pluripotency-associated markers including OCT4 and NANOG, suggesting that both male and female fetal germ cells maintain expression of these genes during and after sexual differentiation of the gonads [55], [56]. The present study raises the question of what is the functional significance of this expression, which will require further investigation. It will also be interesting to study the expression of these markers in more detail in humans and animals where there is no known left-right asymmetry in gonadal development.

### Conclusions

The present study confirms asymmetric distribution of *Cvh/Vasa*-positive germ cells in the embryonic gonads of both sexes. These differences are mirrored by the expression of *ERNI*, a gene associated with the proliferating, self-renewing state of chick embryonic stem cells. However we also uncover asymmetry of other pluripotency-related markers, *PouV/Oct3/4*, *Nanog* and *Sox2*, and find that they do not correlate as well with the distribution of *Cvh-*positive germ cells but also show left-right asymmetry in both sexes.

## Methods

### Eggs and Embryos

All animal experiments were conducted according to UK Home Office guidelines. All embryos were harvested before the 10th day of incubation and the work is therefore exempt from requirement for a licence. Fertilized hens’ (*Gallus gallus*) eggs (Brown Bovan Gold strain) were obtained from Henry Stewart (UK) and incubated at 38°C in a humidified atmosphere for 9 days. Embryos were staged according to Hamburger and Hamilton (H&H) [45]. Chicken embryonic gonads at stage 35 (H&H), when the sex of male and female embryos can be distinguished by morphological appearance of the embryonic gonads, were dissected and fixed with 4% paraformaldehyde in Calcium- and Magnesium-free PBS (pH 7.4) containing 2 mM EGTA at 4°C overnight. A small opening was performed in the part of the attached mesonephroi and dorsal aorta using a fine needle to prevent probe trapping. The fixed embryonic testes and ovaries were then subjected to whole-mount *in situ* hybridization.

### Whole-mount in situ Hybridization and Histology

To generate digoxigenin RNA antisense probes, *cPouV* [38], *cNanog* [38], *cSox2* [39], [57], *ERNI* [41] and *Cvh* [37] plasmids were linearized with *ApaI*, *ApaI*, *PstI*, *KpnI* and *NcoI*, respectively and transcribed with SP6, SP6, T7, T3 and SP6 RNA polymerase, respectively. Whole-mount in situ hybridization and antisense probe preparation were performed as previously described [58]. After in situ hybridization and photography, selected hybridized and post fixed embryonic testes and ovaries were embedded in Fibrowax (BDH^GUN^, UK) for histological sections and then cut on a Zeiss MICROM (Type HM315) microtome at 10 µm thickness.

### Statistical Analysis

To assess the proportion of cells expressing the various genes in embryonic testes and ovaries, expressing cells were counted starting from the first section of the first slide containing gonadal tissue. To avoid counting the same cells more than once, one in three sections were counted until the last section of the gonad was reached. The unpaired Student’s *t*-test with two-tailed distribution and two-sample unequal variance was used to compare (pairwise) the number of expressing cells between left-right sides in male and female embryonic gonads.

## References

[1] Romanoff AL (1960) The avian embryo: structural and functional development. New York: Macmillan. 1305 p.

[2] Smith CA, Sinclair AH (2004) Sex determination: insights from the chicken. Bioessays 26: 120–132. 14745830 10.1002/bies.10400

[3] Smith CA, Sinclair AH (2001) Sex determination in the chicken embryo. J Exp Zool 290: 691–699. 11748617 10.1002/jez.1119

[4] Smith CA, Roeszler KN, Hudson QJ, Sinclair AH (2007) Avian sex determination: what, when and where? Cytogenet Genome Res 117: 165–173. 17675857 10.1159/000103177

[5] Zaccanti F, Vallisneri M, Quaglia A (1990) Early aspects of sex differentiation in the gonads of chick embryos. Differentiation 43: 71–80. 2373289 10.1111/j.1432-0436.1990.tb00432.x

[6] Fujimoto T, Ukeshima A, Kiyofuji R (1976) The origin, migration and morphology of the primordial germ cells in the chick embryo. Anat Rec 185: 139–145. 945007 10.1002/ar.1091850203

[7] Fuyuta M, Miyayama Y, Fujimoto T (1974) Histochemical identification of primordial germ cells in human embryos by PAS reaction. Okajimas Folia Anat Jpn 51: 251–262. 4143063 10.2535/ofaj1936.51.5_251

[8] Smith CA, Katz M, Sinclair AH (2003) DMRT1 is upregulated in the gonads during female-to-male sex reversal in ZW chicken embryos. Biol Reprod 68: 560–570. 12533420 10.1095/biolreprod.102.007294

[9] Smith CA, Roeszler KN, Ohnesorg T, Cummins DM, Farlie PG, et al (2009) The avian Z-linked gene DMRT1 is required for male sex determination in the chicken. Nature 461: 267–271. 19710650 10.1038/nature08298

[10] Kent J, Wheatley SC, Andrews JE, Sinclair AH, Koopman P (1996) A male-specific role for SOX9 in vertebrate sex determination. Development 122: 2813–2822. 8787755 10.1242/dev.122.9.2813

[11] Morais da Silva S, Hacker A, Harley V, Goodfellow P, Swain A, et al (1996) Sox9 expression during gonadal development implies a conserved role for the gene in testis differentiation in mammals and birds. Nature Genetics 14: 62–68. 8782821 10.1038/ng0996-62

[12] Smith CA, Roeszler KN, Sinclair AH (2009) Genetic evidence against a role for W-linked histidine triad nucleotide binding protein (HINTW) in avian sex determination. Int J Dev Biol 53: 59–67. 19123127 10.1387/ijdb.082742cs

[13] Smith CA (2007) Sex determination in birds: HINTs from the W sex chromosome? Sex Dev 1: 279–285. 18391538 10.1159/000108934

[14] Reed KJ, Sinclair AH (2002) FET-1: a novel W-linked, female specific gene up-regulated in the embryonic chicken ovary. Mech Dev 119 Suppl 1S87–S90. 14516666 10.1016/s0925-4773(03)00097-2

[15] Hudson QJ, Smith CA, Sinclair AH (2005) Aromatase inhibition reduces expression of FOXL2 in the embryonic chicken ovary. Dev Dyn 233: 1052–1055. 15830351 10.1002/dvdy.20388

[16] Smith CA, Andrews JE, Sinclair AH (1997) Gonadal sex differentiation in chicken embryos: expression of estrogen receptor and aromatase genes. J Steroid Biochem Mol Biol 60: 295–302. 9219920 10.1016/s0960-0760(96)00196-3

[17] Andrews JE, Smith CA, Sinclair AH (1997) Sites of estrogen receptor and aromatase expression in the chicken embryo. Gen Comp Endocrinol 108: 182–190. 9356214 10.1006/gcen.1997.6978

[18] Zhu L, Marvin MJ, Gardiner A, Lassar AB, Mercola M, et al (1999) Cerberus regulates left-right asymmetry of the embryonic head and heart. Curr Biol 9: 931–938. 10508582 10.1016/s0960-9822(99)80419-9

[19] Levin M (2005) Left-right asymmetry in embryonic development: a comprehensive review. Mech Dev 122: 3–25. 15582774 10.1016/j.mod.2004.08.006

[20] Levin M, Johnson RL, Stern CD, Kuehn M, Tabin C (1995) A molecular pathway determining left-right asymmetry in chick embryogenesis. Cell 82: 803–814. 7671308 10.1016/0092-8674(95)90477-8

[21] Raya A, Izpisua Belmonte JC (2006) Left-right asymmetry in the vertebrate embryo: from early information to higher-level integration. Nature Reviews Genetics 7: 283–293. 10.1038/nrg1830 16543932

[22] Raya A, Izpisua Belmonte JC (2004) Unveiling the establishment of left-right asymmetry in the chick embryo. Mech Dev 121: 1043–1054. 15296970 10.1016/j.mod.2004.05.005

[23] Levin M (1997) Left-right asymmetry in vertebrate embryogenesis. Bioessays 19: 287–296. 9136626 10.1002/bies.950190406

[24] Levin M, Nascone N (1997) Two molecular models of initial left-right asymmetry generation. Med Hypotheses 49: 429–435. 9421811 10.1016/s0306-9877(97)90092-x

[25] Levin M, Pagan S, Roberts DJ, Cooke J, Kuehn MR, et al (1997) Left/right patterning signals and the independent regulation of different aspects of situs in the chick embryo. Dev Biol 189: 57–67. 9281337 10.1006/dbio.1997.8662

[26] Yoshioka H, Meno C, Koshiba K, Sugihara M, Itoh H, et al (1998) Pitx2, a bicoid-type homeobox gene, is involved in a lefty-signaling pathway in determination of left-right asymmetry. Cell 94: 299–305. 9708732 10.1016/s0092-8674(00)81473-7

[27] Rodriguez-Leon J, Rodriguez Esteban C, Marti M, Santiago-Josefat B, Dubova I, et al (2008) Pitx2 regulates gonad morphogenesis. Proc Natl Acad Sci U S A 105: 11242–11247. 18678914 10.1073/pnas.0804904105 PMC2516275

[28] Ishimaru Y, Komatsu T, Kasahara M, Katoh-Fukui Y, Ogawa H, et al (2008) Mechanism of asymmetric ovarian development in chick embryos. Development 135: 677–685. 18199582 10.1242/dev.012856

[29] Guioli S, Lovell-Badge R (2007) PITX2 controls asymmetric gonadal development in both sexes of the chick and can rescue the degeneration of the right ovary. Development 134: 4199–4208. 17959721 10.1242/dev.010249

[30] Hoshino A, Koide M, Ono T, Yasugi S (2005) Sex-specific and left-right asymmetric expression pattern of Bmp7 in the gonad of normal and sex-reversed chicken embryos. Dev Growth Differ 47: 65–74. 15771626 10.1111/j.1440-169x.2004.00783.x

[31] Nakabayashi O, Kikuchi H, Kikuchi T, Mizuno S (1998) Differential expression of genes for aromatase and estrogen receptor during the gonadal development in chicken embryos. J Molec Endocrinol 20: 193–202. 9584834 10.1677/jme.0.0200193

[32] Pain B, Clark ME, Shen M, Nakazawa H, Sakurai M, et al (1996) Long-term in vitro culture and characterisation of avian embryonic stem cells with multiple morphogenetic potentialities. Development 122: 2339–2348. 8756279 10.1242/dev.122.8.2339

[33] van de Lavoir MC, Mather-Love C (2006) Avian embryonic stem cells. Methods Enzymol 418: 38–64. 17141028 10.1016/S0076-6879(06)18003-9

[34] van de Lavoir MC, Mather-Love C, Leighton P, Diamond JH, Heyer BS, et al (2006) High-grade transgenic somatic chimeras from chicken embryonic stem cells. Mech Dev 123: 31–41. 16325380 10.1016/j.mod.2005.10.002

[35] van de Lavoir MC, Diamond JH, Leighton PA, Mather-Love C, Heyer BS, et al (2006) Germline transmission of genetically modified primordial germ cells. Nature 441: 766–769. 16760981 10.1038/nature04831

[36] Park TS, Han JY (2000) Derivation and characterization of pluripotent embryonic germ cells in chicken. Mol Reprod Dev 56: 475–482. 10911397 10.1002/1098-2795(200008)56:4<475::AID-MRD5>3.0.CO;2-M

[37] Lavial F, Acloque H, Bachelard E, Nieto MA, Samarut J, et al (2009) Ectopic expression of Cvh (Chicken Vasa homologue) mediates the reprogramming of chicken embryonic stem cells to a germ cell fate. Dev Biol 330: 73–82. 19324033 10.1016/j.ydbio.2009.03.012

[38] Lavial F, Acloque H, Bertocchini F, Macleod DJ, Boast S, et al (2007) The Oct4 homologue PouV and Nanog regulate pluripotency in chicken embryonic stem cells. Development 134: 3549–3563. 17827181 10.1242/dev.006569

[39] Uwanogho D, Rex M, Cartwright EJ, Pearl G, Healy C, et al (1995) Embryonic expression of the chicken Sox2, Sox3 and Sox11 genes suggests an interactive role in neuronal development. Mech Dev 49: 23–36. 7748786 10.1016/0925-4773(94)00299-3

[40] Rex M, Scotting PJ (1994) Chick HoxB3: deduced amino-acid sequence and embryonic gene expression. Gene 149: 381–382. 7959024 10.1016/0378-1119(94)90183-x

[41] Streit A, Berliner AJ, Papanayotou C, Sirulnik A, Stern CD (2000) Initiation of neural induction by FGF signalling before gastrulation. Nature 406: 74–78. 10894544 10.1038/35017617

[42] Acloque H, Mey A, Birot AM, Gruffat H, Pain B, et al (2004) Transcription factor cCP2 controls gene expression in chicken embryonic stem cells. Nucl Acids Res 32: 2259–2271. 15107494 10.1093/nar/gkh545 PMC407827

[43] Acloque H, Risson V, Birot AM, Kunita R, Pain B, et al (2001) Identification of a new gene family specifically expressed in chicken embryonic stem cells and early embryo. Mech Dev 103: 79–91. 11335114 10.1016/s0925-4773(01)00336-7

[44] Witschi E (1935) Origin of asymmetry in the reproductive system of birds. Am J Anat 56: 119–141.

[45] Hamburger V, Hamilton HL (1951) A series of normal stages in the development of the chick embryo. J Morphol 88: 49–92. 24539719

[46] Shiratori H, Hamada H (2006) The left-right axis in the mouse: from origin to morphology. Development 133: 2095–2104. 16672339 10.1242/dev.02384

[47] Gage PJ, Suh H, Camper SA (1999) Dosage requirement of Pitx2 for development of multiple organs. Development 126: 4643–4651. 10498698 10.1242/dev.126.20.4643

[48] Gage PJ, Suh H, Camper SA (1999) The bicoid-related Pitx gene family in development. Mamm Genome 10: 197–200. 9922405 10.1007/s003359900970

[49] Lin CR, Kioussi C, O’Connell S, Briata P, Szeto D, et al (1999) Pitx2 regulates lung asymmetry, cardiac positioning and pituitary and tooth morphogenesis. Nature 401: 279–282. 10499586 10.1038/45803

[50] Lu MF, Pressman C, Dyer R, Johnson RL, Martin JF (1999) Function of Rieger syndrome gene in left-right asymmetry and craniofacial development. Nature 401: 276–278. 10499585 10.1038/45797

[51] Dubois R, Cuminge D (1978) [Primary asymmetry in the distribution of primordial germ cells during colonization of gonadal buds in chick embryo]. C R Acad Sci Hebd Seances Acad Sci D 286: 535–538. 95895

[52] Dubois R, Cuminge D (1979) [The cause of the primary asymmetry of germ cell distribution in the gonadal primordia of chick embryo]. C R Acad Sci Hebd Seances Acad Sci D 288: 895–898. 111830

[53] Dubois R, Cuminge D (1978) [Statistical study of the primary asymmetry in primordial germ cells distribution in the chick embryo]. C R Acad Sci Hebd Seances Acad Sci D 286: 1613–1616. 97018

[54] Swartz WJ, Domm LV (1972) A study on division of primordial germ cells in the early chick embryo. Am J Anat 135: 51–70. 5069145 10.1002/aja.1001350106

[55] Kerr CL, Hill CM, Blumenthal PD, Gearhart JD (2008) Expression of pluripotent stem cell markers in the human fetal ovary. Hum Reprod 23: 589–599. 18203707 10.1093/humrep/dem411

[56] Kerr CL, Hill CM, Blumenthal PD, Gearhart JD (2008) Expression of pluripotent stem cell markers in the human fetal testis. Stem Cells 26: 412–421. 18024420 10.1634/stemcells.2007-0605

[57] Rex M, Uwanogho DA, Orme A, Scotting PJ, Sharpe PT (1997) cSox21 exhibits a complex and dynamic pattern of transcription during embryonic development of the chick central nervous system. Mech Dev 66: 39–53. 9376322 10.1016/s0925-4773(97)00086-5

[58] Stern CD (1998) Detection of multiple gene products simultaneously by in situ hybridization and immunohistochemistry in whole mounts of avian embryos. Curr Top Dev Biol 36: 223–243. 9342531 10.1016/s0070-2153(08)60505-0

